# Epidemiology of tuberculosis in Papua New Guinea: analysis of case notification and treatment-outcome data, 2008–2016

**DOI:** 10.5365/wpsar.2018.9.1.006

**Published:** 2018-06-15

**Authors:** Paul Aia, Lungten Wangchuk, Fukushi Morishita, Jacob Kisomb, Robin Yasi, Margaret Kal, Tauhid Islam

**Affiliations:** a National Department of Health, Papua New Guinea.; b World Health Organization Representative Office for Papua New Guinea.; c World Health Organization Regional Office for the Western Pacific.

## Abstract

Papua New Guinea has strengthened its surveillance system for tuberculosis (TB) under the National TB Program. This paper provides an overview of TB surveillance data at the national and subnational levels from 2008 to 2016.

TB case notification has consistently increased since 2008 with 6184 cases (93 per 100 000 population) in 2008 to 28 598 (359 per 100 000 population) in 2014 and has stabilized since 2014 with 28 244 cases (333 per 100 000 population) in 2016. The population-screening rate for TB rose from 0.1% in 2008 to 0.4% in 2016. Notified cases were dominated by extra-pulmonary TB (EP-TB, 42.4% of all cases in 2016). The proportion of pulmonary TB cases with no sputum test results was high with a national average of 26.6%. The regional variation of case notifications was significant: the Southern Region had the highest number and rate of notified TB cases. Of the nationally reported cases, 26.7% occurred in children. Treatment success rates remained low at 73% for bacteriologically confirmed TB and 64% for all forms of TB in 2016, far below the global target of 90%. For all forms of TB, 19% of patients were lost to follow-up from treatment.

An analysis of TB data from the national surveillance system has highlighted critical areas for improvement. A low population-screening rate, a high proportion of pulmonary TB cases without sputum test results and a low treatment success rate suggest areas for improvement in the National TB Program. Our additional subnational analysis helps identify geographical and programmatic areas that need strengthening and should be further promoted to guide the programme’s direction in Papua New Guinea.

**Epidemiology of tuberculosis in Papua New Guinea: analysis of case notification and treatment-outcome data, 2008–2016**

## Introduction

Papua New Guinea has high burdens of tuberculosis (TB), multidrug-resistant TB (MDR-TB) and TB/HIV co-infection. (*1*) The estimated TB incidence in Papua New Guinea in 2016 was 432 cases per 100 000 population. (*1*)

Papua New Guinea initiated directly observed treatment, short-course (DOTS), a global TB control strategy, in 2008. While other countries have adopted newer global strategies, Papua New Guinea is facing challenges in adapting and implementing basic DOTS. With external support, DOTS was expanded nationwide, and the standardized-routine-surveillance system was strengthened, resulting in the capturing of TB reports nationwide since 2012.

This paper provides an overview of national and subnational TB surveillance data in Papua New Guinea, from the inception of the DOTS strategy in 2008 to 2016. The results are expected to facilitate better understanding of TB epidemiology in Papua New Guinea, help identify programmatic gaps and inform actions.

## Methods

We conducted a retrospective descriptive analysis of TB cases and treatment outcomes using routine surveillance data from the national TB database for the period 2008–2016. TB laboratory results, case notifications, HIV testing results and treatment outcomes were analysed by disease category, geographic areas and demographic variables. Papua New Guinea has a decentralized health-care system; TB services are delivered by provincial and local governments under policies set by the National Department of Health. (*2*) The National TB Program defined a basic management unit (BMU) as the initial point of TB data collection. There are approximately 275 BMUs and 114 laboratories with TB testing capacities with varying catchment populations across 22 provinces. Recording and reporting formats are in line with WHO recommendations. (*3*) The BMU reports are consolidated into a standardized report that is submitted quarterly to the provincial health office and to the National TB Program at the National Department of Health. The aggregated national database is maintained in Excel.

We obtained case-notification and treatment-outcome data from the aggregated national database. Population data were projected using 2000 and 2011 census data; (*4*, *5*) age- and sex-disaggregated population data for 2015 were sourced from LivePopulation.com. (*6*) To assess case-finding efforts, we calculated a population-screening rate, (*7*) which we defined as the number of people with presumptive TB examined by smear microscopy divided by the total population in each year. The smear-positivity rate was defined as the number of smear-positive patients divided by the total number of people examined for TB. Treatment outcomes were classified as per WHO definitions as cured, treatment completed, treatment failed, died, lost to follow-up and not evaluated. Treatment success was defined as the sum of cured and treatment completed. (*3*) R version 3.4.1 was used for data analysis and visualization. QGIS version 2.18 was used to produce maps.

### Ethics statements

As this report used routinely available data and no personal identifying information was collected, ethical clearance was not required according to local regulations.

## Results

**Fig. 1:**
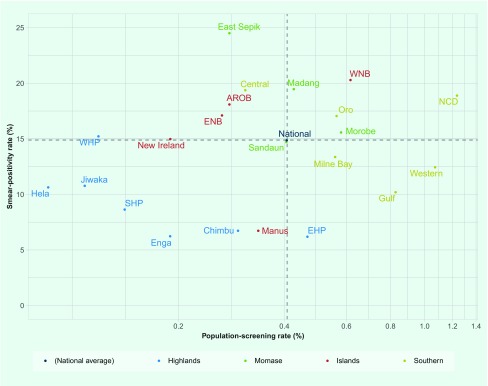
Population-screening rate vs smear-positivity rate by province, Papua New Guinea, 2016

Note: A log scale was used for the *y*-axis. AROB: Autonomous Region of Bougainville, EHP: Eastern Highlands Province, ENB: East New Britain, NCD: National Capital District, SHP: Southern Highlands Province, WHP: Western Highlands Province, WNB: West New Britain.

**Fig. 2:**
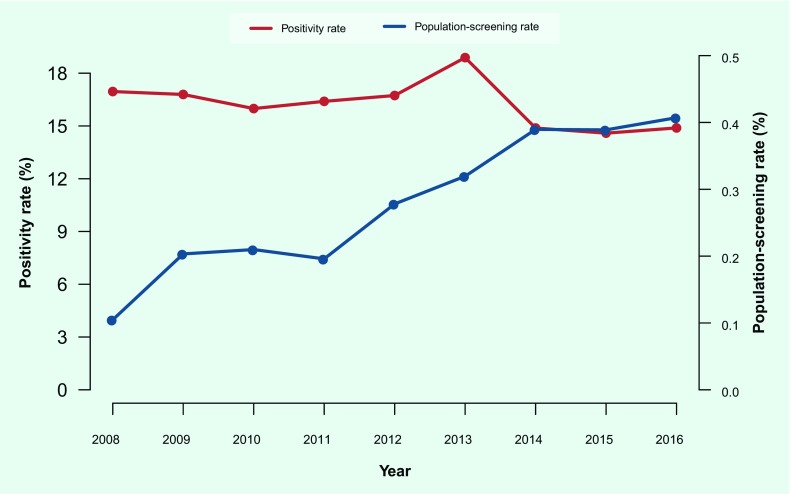
Population-screening rate vs smear-positivity rate, Papua New Guinea, 2008–2016

In 2016, 0.4% of the national population was screened for TB, and 15% of those screened were smear positive; the percentages varied across regions and provinces (**Fig. 1**). Low levels of both indicators were observed in the Highlands Region (0.22% screened, 7.6% positive), low screening with high positivity was observed in the Islands Region (0.34% screened, 17.9% positive), and moderate screening with high positivity was observed in the Momase Region (0.44% screened, 17.9% positive) and the Southern Region (0.78% screened, 15.8% positive). While national population screening increased over time, from 0.1% in 2008 to 0.4% in 2016, smear positivity did not decrease proportionately (17% in 2008 and 15% in 2016) (**Fig. 2**).

**Fig. 3:**
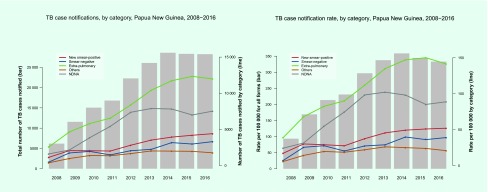
TB case notification (absolute number and rate) by diagnostic category, Papua New Guinea, 2016

In 2016, the case notification rate for all forms of TB was 333 per 100 000 population (*n* = 28 244). The number and rate of case notifications of all forms of TB increased during 2008–2014 but stabilized during 2015–2016 (**Fig. 3**). The total TB caseload was driven mainly by extra pulmonary TB (EP-TB) (*n* = 11 984, 42% in 2016) and pulmonary TB cases without sputum test results (either the test was not done or the result was not available) (*n* = 7527, 27% in 2016). Among all TB cases and pulmonary TB cases, 15.6% and 25.9% were bacteriologically confirmed, respectively.

NDNA: Sputum smear testing not done or results are not available

**Fig. 4:**
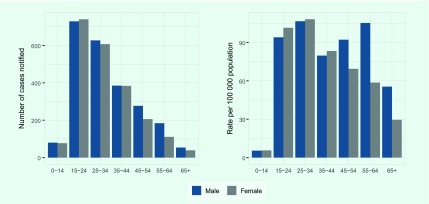
TB case notification (new smear-positive) by age and sex, Papua New Guinea, 2016

Case notification of new smear-positive TB was highest in the 15–24-year-old age group (**Fig. 4**). Case notification rates show two peaks in the 25–34-year old age group (for both males and females) and in the 55–64-age group (for males only). Case notification rates were equally high in younger males and females (15–34 years old), whereas higher rates were observed in men in older age groups.

The age- and sex-disaggregated population data in 2015 were used to calculate case notification rates for 2016.

**Fig. 5:**
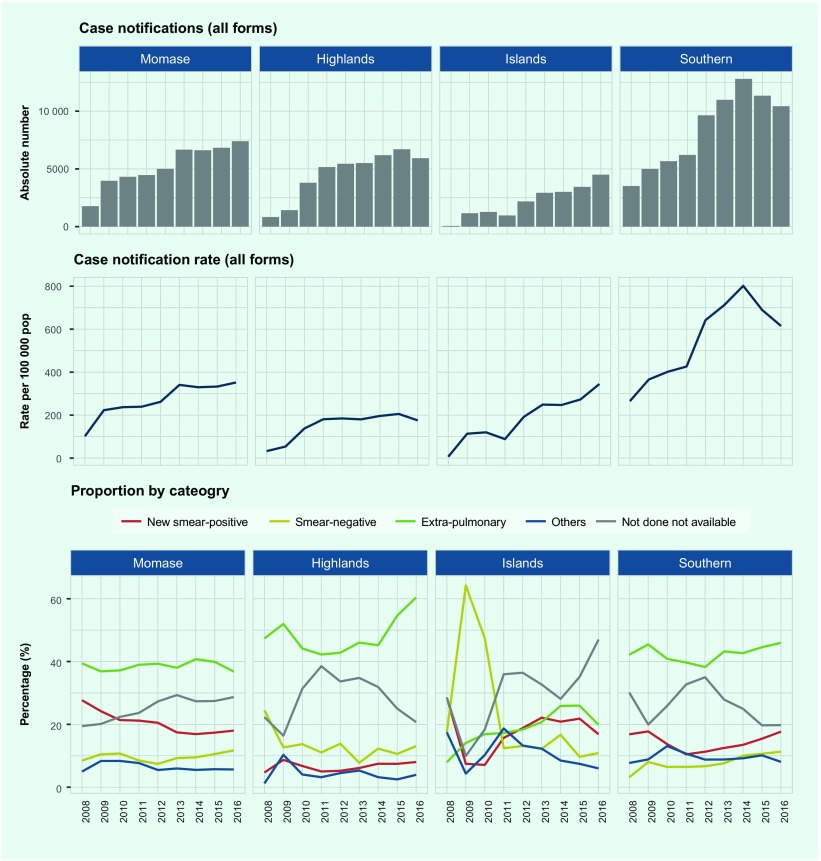
TB case notification (absolute number, rate and percentage) by region, Papua New Guinea, 2008–2016

We observed variations in case notification among the four regions (**Fig. 5** and **6A**). The Southern Region had the highest rate (615 per 100 000 population) in 2016, with an increasing annual trend since 2008 and a peak in 2014 (802 per 100 000 population). The rate in the regions of Momase and Islands increased during the study period to over 300 per 100 000 population in 2016, while the rate in the Highlands plateaued at around 200 per 100 000 population in 2013. EP-TB was the main contributor to the overall case notifications in the Momase, Highlands and Southern regions during 2016 (37%, 60% and 46%, respectively), followed by pulmonary TB cases without sputum test results (29%, 21% and 20%, respectively). The proportion of pulmonary TB cases without sputum test results declined in the Highlands Region (from 39% in 2011 to 21% in 2016) and the Southern Region (from 35% in 2012 to 20% in 2016). In the Islands Region, pulmonary TB cases without sputum test results were most commonly reported, and they sharply increased from 28% in 2014 to 47% in 2016. In all regions, the proportion of new smear-positive TB cases remains low at below 20%.

**Fig. 6A:**
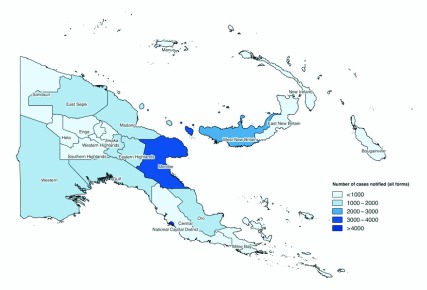
Number of TB case notifications by province, Papua New Guinea, 2016

At the provincial level, high case notification rates of more than 600 per 100 000 population were reported in the National Capital District (NCD), Western, Gulf and West New Britain provinces in 2016 (**Fig. 6B**). Ten provinces contributed to 76% of the reported TB burden: NCD, Western, Gulf, Oro, East Sepik, Madang, Morobe, Eastern Highlands, Chimbu, and West New Britain.

**Fig. 6B:**
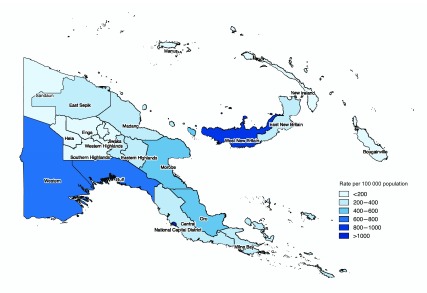
TB case notification rate by province, Papua New Guinea, 2016

**Fig. 7:**
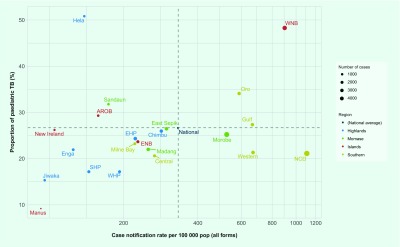
Proportion of paediatric TB among all notified TB cases by province, Papua New Guinea, 2016

Note: A log scale was used for the *x*-axis. AROB: Autonomous Region of Bougainville, EHP: Eastern Highlands Province, ENB: East New Britain, NCD: National Capital District, SHP: Southern Highlands Province, WHP: Western Highlands Province, WNB: West New Britain

Paediatric TB cases (age ≤ 14 years old) constituted 26.7% (*n* = 7541) of all notified TB cases in 2016. Most of the provinces reported proportions of paediatric TB cases between 20% and 30% (**Fig. 7**). Four provinces—Manus, Jiwaka, Southern Highlands and Western Highlands—reported proportions of paediatric cases of less than 20%. Four provinces—Sandaun, Hela, Oro and West New Britain—reported proportions of paediatric cases of more than 30%; particularly high proportions were reported in Hela (51%) and West New Britain (48%) (**Fig. 7**).

In 2016, the proportion of pulmonary TB among total TB notifications was 27.3% nationally. New smear-positive cases accounted for 15.6% of TB notifications nationwide, with the lowest proportion found in the Highlands Region (8%) (**Table 1**). EP-TB contributed 42.4% of the total notifications in 2016, with the highest proportion reported in the Highlands Region (60.4%). The proportions of pulmonary TB cases without sputum test results ranged from 19.8% in the Southern Region to 47% in the Islands Region as compared to the national average of 26.6%.

**Table 1:** Summary indicators for TB programme, Papua New Guinea, 2016

Region	Province	Case notification (all forms)		Category of TB					Paediatric cases (%)	TB patients tested for HIV (%)	HIV-positive among patients tested (%)
		Number	Rate per 100 000 population	New smear-positive (%)	Smear-negative (%)	Extra-pulmonary (%)	Others (%)	Not done not available (%)			
Momase		7393	352	18.0	11.7	36.8	5.6	28.7	25.4	45	5.3
-	**East Sepik**	**1532**	**301**	**16.4**	**5.0**	**32.1**	**4.4**	**42.0**	**26.6**	**9.6**	**9.5**
	**Madang**	**1430**	**253**	**23.7**	**16.6**	**38.8**	**5.9**	**15.3**	**22.1**	**36.6**	**5.2**
	**Morobe**	**3938**	**529**	**15.9**	**12.7**	**38.1**	**6.0**	**28.7**	**25.4**	**63.6**	**5.4**
	**Sandaun**	**493**	**175**	**23.1**	**10.1**	**34.9**	**6.1**	**26**	**31.8**	**31.2**	**0.0**
**Highlands**		**5924**	**175**	**8.0**	**13.1**	**60.4**	**3.9**	**20.7**	**23.6**	**45.7**	**8.9**
-	**Chimbu**	**1274**	**286**	**5.7**	**15.7**	**44.4**	**4.9**	**30.9**	**26.1**	**22.9**	**12.7**
	**Eastern Highlands**	**1481**	**225**	**8.4**	**13.8**	**78.5**	**3.4**	**18.2**	**24.4**	**33.6**	**5.0**
	**Enga**	**645**	**126**	**8.8**	**12.2**	**63.6**	**0.3**	**16.1**	**22.0**	**82.3**	**14.9**
	**Hela**	**397**	**139**	**5.5**	**9.8**	**74.1**	**2.3**	**8.3**	**50.9**	**34.3**	**5.9**
	**Jiwaka**	**435**	**96**	**10.6**	**14.7**	**62.5**	**3.9**	**8.3**	**15.4**	**86.4**	**4.0**
	**Southern Highlands**	**869**	**145**	**8.2**	**13.5**	**46.8**	**4.3**	**28.0**	**17.3**	**57.1**	**7.9**
	**Western Highlands**	**823**	**194**	**10.0**	**8.5**	**56.7**	**6.8**	**18.0**	**17.3**	**46.2**	**9.7**
**Islands**		**4502**	**345**	**16.8**	**10.9**	**19.9**	**6.0**	**47**	**39.7**	**11.4**	**8.2**
-	**Bougainville**	**463**	**159**	**28.1**	**6.3**	**27.6**	**6.9**	**31.7**	**29.4**	**8.9**	**4.9**
	**East New Britain**	**901**	**230**	**21.6**	**18.2**	**25**	**7.5**	**30.1**	**23.8**	**24.4**	**15.0**
	**Manus**	**65**	**93**	**24.6**	**12.3**	**33.8**	**10.8**	**18.5**	**9.2**	**10.8**	**28.6**
	**New Ireland**	**255**	**105**	**25.9**	**15.3**	**35.3**	**6.3**	**17.3**	**26.3**	**47.8**	**0.8**
	**West New Britain**	**2818**	**907**	**12.5**	**8.9**	**15.2**	**5.2**	**58.3**	**48.4**	**4.4**	**3.2**
**Southern**		**10 425**	**615**	**17.7**	**11.3**	**45.9**	**8.1**	**19.8**	**23.7**	**31.3**	**7.3**
-	**Central**	**860**	**268**	**22.7**	**6.0**	**43.8**	**9.2**	**20.9**	**20.7**	**3.0**	**19.2**
	**Gulf**	**1264**	**669**	**14.2**	**11.6**	**43.7**	**10**	**20.5**	**27.5**	**33.9**	**5.4**
	**Milne Bay**	**699**	**223**	**28.3**	**10.7**	**32.9**	**8.0**	**21.6**	**23.3**	**19.3**	**8.9**
	**National Capital District**	**4783**	**1117**	**17.5**	**12.5**	**42.8**	**8.0**	**24.4**	**21.3**	**42.5**	**7.6**
	**Oro**	**1284**	**592**	**15.1**	**9.1**	**49.1**	**8.8**	**17.9**	**34.2**	**20.6**	**10.6**
	**Western**	**1535**	**674**	**15.4**	**12.3**	**62.1**	**5.3**	**4.8**	**21.4**	**24.7**	**4.2**
**National**		**28 244**	**333**	**15.6**	**11.7**	**42.4**	**6.2**	**26.6**	**26.7**	**34.8**	**7.1**

Of all TB cases, 34.8% were tested for HIV with variations at the subnational level ranging from 3% in Central Province to 86.4% in Jiwaka Province (**Table 1**). The regional testing rate was highest in the Highlands (45.7%), with two of its provinces (Enga and Jiwaka) achieving an HIV testing rate of ≥ 80%. The HIV testing rate was the lowest in the Islands (11.4%). In 2016, 7.1% of the notified TB patients who were tested for HIV were HIV positive. HIV positivity ranged from 0% in Sandaun Province to 28.6% in Manus Province. Six provinces had HIV positivity rates of 10% or more: Chimbu, Enga, East New Britain, Manus, Central and Oro (**Table 1**).

**Fig. 8:**
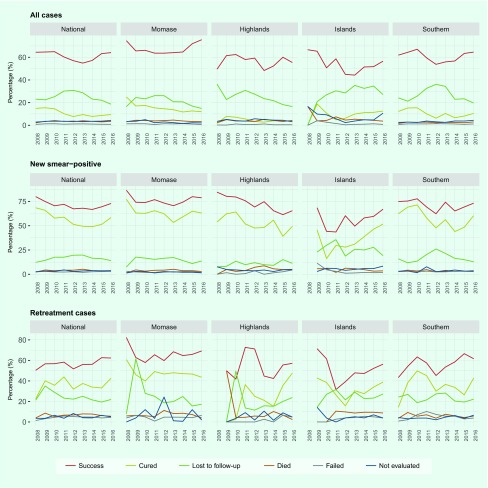
Treatment outcomes at national and regional levels, Papua New Guinea, 2008–2016

The treatment success rate for all TB cases at the national level remained low in comparison to the global standard, (*1*) ranging between 55% and 65% during the study period (**Fig. 8**). The percentage of patients lost to follow-up declined over time; nevertheless, it remained high at ≥ 19% in 2016. Loss to follow-up and not evaluated were the major contributing factors towards a low treatment success rate in Papua New Guinea. In 2016, 986 deaths were reported. Loss to follow-up remained a major issue for all regions, with the highest rate in the Islands (27% in 2016). We observed higher treatment success rates in new smear-positive cases (73% in 2016), though the cure rate remained considerably lower than the treatment success rate.

## Discussion

In this paper, we report national and subnational TB surveillance data that provide an overview of the TB epidemiological and programmatic situation in Papua New Guinea over nine years. From 2008 to 2012, the country succeeded in expanding DOTS and strengthening the national surveillance system to capture nationwide data. Improved case-finding efforts resulted in a doubling of population screening from 0.2% in 2011 to 0.4% in 2014. The population-screening rate has remained static since 2014, and the rate was found to be low compared to other countries such as Cambodia (1.1% in 2013) (*7*) and Tajikistan (0.57% in 2013). (*8*) The programme in Papua New Guinea might not be reaching hard-to-reach populations possibly due to the policy to focus on a limited number of health centres. (*9*)

In the setting of an effective TB programme, the smear-positivity rate is inversely proportional to the population-screening rate. (*7*) In Cambodia, the smear-positivity rate declined from 29% in 2001 to 8% in 2013 along with an increased screening rate. (*7*) In contrast, the smear-positivity rate in Papua New Guinea did not decrease considerably, indicating that improved case detection had a limited impact on reducing infectiousness or that only highly presumptive cases were tested, leading to missed cases. Delayed diagnoses (due to limited access to health facilities and microscopy centres) and low treatment success rates likely contribute to high smear positivity. Papua New Guinea has only 114 microscopy facilities across 275 BMUs with weak referral systems, resulting in overreliance on clinical diagnosis. These health systems gaps can also account for the high proportion of pulmonary TB cases without sputum test results (26.6%) and the low proportion of bacteriologically confirmed TB among pulmonary TB cases (25.9%) compared to 38% in the Western Pacific Region and 57% globally. (*1*)

In parallel with increased population-screening rates, case notification rates steadily increased from 2008 to 2014, spiked in 2012 and plateaued in 2014. The highest case notification rates were in the 15–64-year-old age group, diverging from the pattern of highest case notification rates in older populations seen in most high-burden countries in the Western Pacific Region. (*10*) Similarly, the proportion of paediatric TB in Papua New Guinea (26.7%) was found to be higher than other high-burden countries in the Western Pacific Region. (*11*) Paediatric TB represents recent transmission and can be a sentinel marker of disease transmission. (*12*, *13*) Although over-diagnosis is possible, the large proportion of paediatric TB cases indicates ongoing community transmission. We believe that this calls for improvements in early case finding and appropriate community preventive measures, including contact investigation.

Without additional information, we cannot determine the causes of regional variations in TB case notification. The increased case notification rate in the Southern Region might reflect might reflect increased true TB incidence or improved programme activities, or both. Given Papua New Guinea’s rich regional sociocultural diversity, different factors can affect an individual’s TB risk and health-seeking behaviours as well as a programme’s performance measures in different ways. Ultimately, these differences may lead to regional differences in case notification rates.

The high proportion of pulmonary TB cases without sputum test results is a major barrier in understanding TB epidemiology in Papua New Guinea. Many factors could have contributed to this high proportion, including limited accessibility to a TB laboratory and unreliable sputum transport systems. (*14*) Without increasing the number of quality-assured functional TB laboratories, the challenge to increase bacteriological confirmation of TB will remain.

Despite the national mandate to test everyone diagnosed with TB for HIV infection, only 34.8% of TB patients were tested; 7% were positive, a percentage comparable to other high-risk populations, such as sex workers (14.9%), men who have sex with men and transgender individuals (8.5%). (*15*) Among other countries in the Western Pacific Region with a high burden of TB, the percentage of TB patients tested for HIV ranged from 13% in the Philippines to 84% in Cambodia; HIV positivity ranged from < 1% in the Philippines to 4% in Cambodia. (*1*) While the percentage of TB patients tested for HIV in Papua New Guinea is comparable with other high-burden countries in the Region, positivity is higher. Collaboration between TB and HIV programmes and the implementation of integrated service delivery models with proper monitoring are essential to reduce the burden of TB/HIV co-infection.

Treatment success did not improve during the study period; the national average remained around 65%, far below the global target of 90%, (*16*) and the Western Pacific Region rate of more than 85%. (*1*, *10*) None of the regions achieved the > 85% treatment success target. Loss to follow-up continues to be a major challenge that has likely resulted in an underreporting of deaths. New smear-positive cases had better outcomes compared to re-treatment cases but did not reach the global target. To improve treatment outcomes, further action is needed to strengthen patient support, including daily treatment, monitoring, counselling and continued efforts to strengthen the health system and address socioeconomic and physical barriers to accessing TB services. (*17*) Family DOT and self-administration are currently practised in Papua New Guinea but have not led to improved treatment success rates. Revisiting the care modality by strengthening community involvement and using an informal health workforce for treatment support is warranted. To improve access to TB treatment, it is essential not only to increase the number of BMUs but also to advance integrated service delivery through the full network of public health facilities, including aid posts (lowest-level public health facility) in communities.

This report has several limitations. An analysis of drug-resistant (DR-TB) was not included because a nationwide data collection system for DR-TB has not been established. Laboratory data used in the analysis were limited to smear microscopy results since data on GeneXpert and culture tests were not captured in the surveillance system. Data quality might have been an issue, especially as the surveillance system was being established, and the reporting rate was low between 2008 and 2012. Hence, trends in those years should be interpreted with caution. In addition, a high percentage of pulmonary TB cases without sputum test results hindered the interpretation of results.

Despite these limitations, we have provided an overview of TB surveillance data and identified patterns in TB epidemiology and programmatic performance in Papua New Guinea. In particular, subnational-level analysis helped identify geographical and programmatic areas that can be prioritized for improvement. (*7*, *18*) The use of subnational data should be further strengthened and routinely performed for operational planning and implementation of effective TB programmes in Papua New Guinea.
